# MAST4 controls cell cycle in spermatogonial stem cells

**DOI:** 10.1111/cpr.13390

**Published:** 2023-01-02

**Authors:** Seung‐Jun Lee, Ka‐Hwa Kim, Dong‐Joon Lee, Pyunggang Kim, Jinah Park, Seong‐Jin Kim, Han‐Sung Jung

**Affiliations:** ^1^ Division in Anatomy and Developmental Biology, Department of Oral Biology, Taste Research Center Oral Science Research Center, BK21 FOUR Project, Yonsei University College of Dentistry Seoul South Korea; ^2^ Department of MAST Research Division in GILO Research Institute, GILO Foundation Seoul South Korea; ^3^ Division in Research Institute Laboratory of Musculoskeletal Research, Medpacto Inc. Seoul South Korea

## Abstract

Spermatogonial stem cell (SSC) self‐renewal is regulated by reciprocal interactions between Sertoli cells and SSCs in the testis. In a previous study, microtubule‐associated serine/threonine kinase 4 (MAST4) has been studied in Sertoli cells as a regulator of SSC self‐renewal. The present study focused on the mechanism by which MAST4 in Sertoli cells transmits the signal and regulates SSCs, especially cell cycle regulation. The expression of PLZF, CDK2 and PLZF target genes was examined in WT and *Mast4* KO testes by Immunohistochemistry, RT‐qPCR and western blot. In addition, IdU and BrdU were injected into WT and *Mast4* KO mice and cell cycle of SSCs was analysed. Finally, the testis tissues were cultured in vitro to examine the regulation of cell cycle by MAST4 pathway. *Mast4* KO mice showed infertility with Sertoli cell‐only syndrome and reduced sperm count. Furthermore, *Mast4* deletion led to decreased PLZF expression and cell cycle progression in the testes. MAST4 also induced cyclin‐dependent kinase 2 (CDK2) to phosphorylate PLZF and activated PLZF suppressed the transcriptional levels of genes related to cell cycle arrest, leading SSCs to remain stem cell state. MAST4 is essential for maintaining cell cycle in SSCs via the CDK2‐PLZF interaction. These results demonstrate the pivotal role of MAST4 regulating cell cycle of SSCs and the significance of spermatogenesis.

## Graphical abstract

MAST4 maintains cell cycle and self‐renewal in spermatogonial stem cells. In Sertoli cells, FGF2 induces MAST4 to promote ERM transcription factor and *Cxcl12* transcription, and to transmit signals to spermatogonial stem cells. Activated CDK2 phosphorylates PLZF and suppresses the transcription of PLZF target genes related to cell cycle arrest in spermatogonial stem cells. However, *Mast4* KO does not proceed this mechanism, resulting in cell cycle arrest in spermatogonial stem cells. FGF2 and CXCL12, which are upstream and downstream of MAST4, could rescue cell cycle arrest and enable spermatogonial stem cells to maintain their cell cycle.

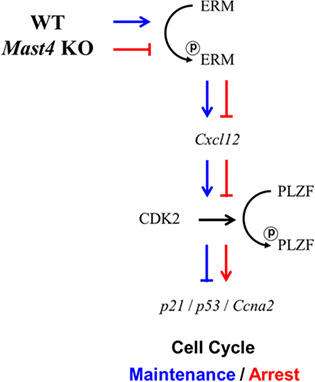

## 1 INTRODUCTION

Stem cells are characterized by their ability to self‐renew and maintain their pool, remaining primarily quiescent in the body.^1^ Self‐renewal is the process by which stem cells divide to make more stem cells, maintaining the stem cell pool throughout life. This requires cell cycle control and cell cycle has been proposed as a gatekeeper for self‐renewal.^2^ Dynamic changes in gene expression as a function of cell cycle progression are regulated by activities of specific cyclins and cyclin‐dependent kinases (CDKs).^3^ In the intestine, Wnt/β‐catenin pathway upregulates nuclear translocation of β‐catenin and promotes the expression of Wnt target genes, such as *Ccnd1*, *Myc* and *Lgr5*, which are associated with the self‐renewal of intestinal stem cells.^4^, ^5^ In pancreatic β cells, cyclin D2 is required for postnatal β cell self‐renewal in mice and the overexpression of cyclin D2 increases the self‐renewal and cell mass of β cells.^6^, ^7^ Furthermore, BMI is required for the self‐renewal of adult lung stem cells and multiple homeobox genes, such as cyclin‐dependent kinase inhibitor 2b (*p15*), and 1c (*p57*), are increased in *Bmi1* mutant lung cells.^8^, ^9^ Although several studies have reported stem cell self‐renewal and maintenance, the molecular regulation about cell cycle progression of stem cells remains unclear.

Spermatogenesis originates in spermatogonial stem cells (SSCs), which are the only stem cells in the male germline that undergo self‐renewal and division.^10^ SSCs give rise to undifferentiated spermatogonia, which undergo differentiation and further rounds of division to produce spermatocytes that enter the meiotic process, thereby supporting daily sperm production.^11^, ^12^ Spermatogenesis, supported by the self‐renewal and differentiation of SSCs, is strictly controlled by a special microenvironment in the seminiferous tubules.^13^ Sertoli cells, the only somatic cell type in the tubules, directly interact with SSCs to control their proliferation and differentiation through the secretion of specific factors such as glial cell line‐derived neurotrophic factor and ETS variant 5 transcription factor (also known as ERM).^13^, ^14^, ^15^, ^16^ Although the foundation for SSC studies has been established based on morphological analyses,^17^, ^18^, ^19^ it is vital to investigate relevant core concepts through molecular mechanisms involving cell–cell interactions or intracellular signalling pathways such as cell cycle regulation.

Promyelocytic leukaemia zinc finger (PLZF) protein belongs to the Krüppel‐like zinc finger protein family, which is involved in the regulation of diverse cellular processes, including cell proliferation, apoptosis, differentiation and development.^20^ PLZF is a negative regulator of cell division during embryogenesis,^21^ and it is essential for the osteogenic differentiation of human mesenchymal stem cells during development. Furthermore, PLZF augments SSC self‐renewal in the testes and the deletion of *Plzf* results in infertility.^22^ PLZF, a zinc finger transcription factor, controls the expression of lineage‐specific target genes, such as *p21*, *p53*, *Ccna2* and *c‐Myc*,^23^, ^24^, ^25^ thereby instructing stem/progenitor cells to accept certain cell fate programs for self‐renewal or differentiation.

Our previous study has shown that microtubule‐associated serine/threonine kinase 4 (MAST4) regulates SSC self‐renewal via the FGF2/ERM pathway.^26^ The mechanism by which MAST4 regulates SSC self‐renewal has been investigated in Sertoli cells intensively. Therefore, the purpose of this study is to investigate the effect of MAST4 on SSCs and how MAST4 regulates cell cycle of SSCs. Our results indicated that PLZF expression and cell cycle were decreased in *Mast4* knockout (KO) testes at postnatal week 22 (PN 22W). MAST4 induced CDK2 to phosphorylate PLZF, and activated PLZF suppressed the transcription of *p21*, *p53* and *Ccna2*, allowing SSCs to remain in the stem cell state. Furthermore, FGF2/CXCL12 treatment in in vitro culture rescued cell cycle of *Mast4* KO SSCs, suggesting that MAST4 plays a role in the maintenance of SSCs. To sum it up, these results demonstrate a novel mechanism of spermatogenesis by MAST4 regulating cell cycle of SSCs.

## 2 MATERIALS AND METHODS

### 2.1 Animals

All animal experiments were approved by Yonsei University Health System Institutional Animal Care and Use Committee (YUHS‐IACUC) in accordance with the Guide for the care and use of laboratory animal (National Research Council, USA). The animal study plan for these experiments (2021‐0093) was reviewed and approved by this committee. And all experiments were performed in accordance with the guidelines of this committee. Mice were housed in a temperature‐controlled room (22°C) under artificial illumination (lights on from 05:00 to 17:00) and 55% relative humidity, and they had ad libitum access to food and water. All the operational procedures were performed under deep anaesthesia. To generate *Mast4* KO mice by CRISPR/Cas9‐mediated gene targeting was described previously.^26^

### 2.2 Sperm count

To count sperm, both cauda epididymides from each mouse were collected, dissected and placed in 2 ml of Hanks' Balanced Salt Solution (HBSS; #14025‐092, Life Technologies, USA) for 30 min to allow the release of motile cells (swim‐out procedure). The total sperm number from suspension sperm was obtained using a haemocytometer.

### 2.3 Immunohistochemistry

Samples were fixed in 4% paraformaldehyde in phosphate buffered saline (PBS) and then embedded in paraffin using standard procedures. Sections (4‐μm thickness) of the specimens were boiled in 10 mM citrate buffer (pH 6.0) and cooled at room temperature for 20 min. The specimens were incubated with primary antibodies at 4°C overnight. Primary antibodies are listed in Table S1. The specimens were incubated with Alexa Flour secondary antibodies (Invitrogen, OR, USA; 1:200) for 2 h at room temperature and were counterstained with DAPI (D1306, Invitrogen, OR, USA; 1:1000). The sections were examined using a confocal laser microscope (TCS SP8, Leica, Germany).

### 2.4 IdU/BrdU injection and cell cycle time (*T*_*c*_) calculation

The 5′‐iodo‐2′‐deoxyuridine (IdU)/5′‐bromo‐2′‐deoxyuridine (BrdU) injection for calculating *T*_*c*_ is described schematically in Figure S1. Both IdU and BrdU were injected into the mice (100 mg/kg). Testes were embedded in paraffin and serially sectioned into 4‐μm slices. The specimens were incubated with anti‐PLZF (SC‐28319, Santa Cruz Biotechnology, Inc., USA; 1:200) to label SSCs in serial sections. In addition, the specimens were incubated with anti‐BrdU (#347580, BD Biosciences, USA; 1:100), which recognizes both IdU and BrdU, and anti‐BrdU (ab6326, Abcam, UK; 1:200) for double staining. *T*_*c*_ was calculated using the ratio of IdU‐only‐labelled cells (leaving cells, L_cells_) and PLZF‐labelled cells (the total number of SSCs, P_cells_).^27^, ^28^ Cells labelled with IdU only left the S phase during the interval time (12 h) between the IdU and BrdU injections. Therefore, the following formula can be used to calculate *T*_*c*_:

$$\frac{T_{c}}{\text{Interval time}}=\frac{P_{\text{cells}}}{L_{\text{cells}}}\;\text{or}\;T_{c}=\frac{P_{\text{cells}}}{L_{\text{cells}}}\times \text{interval time}.$$

### 2.5 RT‐qPCR

The total RNA was extracted using TRIzol® reagent (#15596‐026, Thermo Fisher Scientific, USA). The extracts were reverse transcribed using Maxime RT PreMix (#25081, iNtRON, Korea). RT‐qPCR was performed using a StepOnePlus Real‐Time PCR System (Applied BioSystems, USA). The amplification program consisted of 40 cycles of denaturation at 95°C for 15 s and annealing at 62°C for 30 s. The expression levels of each gene are expressed as normalized ratios against the *B2m* housekeeping gene. Primers are listed in Table S2.

### 2.6 Western blot

Cell extracts from testis tissues were fractionated by SDS‐PAGE and transferred to a polyvinylidene difluoride membrane (PVDF; Millipore) using a transfer apparatus according to the manufacturer's protocols. After incubation with 5% skim milk in TBST (10 mM Tris, pH 7.4, 150 mM NaCl, 0.1% Tween 20) for 1 h, the membrane was incubated with primary antibodies at 4°C overnight. Membranes were washed three times for 10 min and incubated with HRP‐conjugated secondary antibodies for 2 h. Blots were washed three times with TBST and developed with the ECL system (RPN2232, GE Healthcare Life Sciences, USA) according to the manufacturer's protocols. Primary antibodies are listed in Table S1.

### 2.7 Immunoprecipitation (IP)

Flag‐PLZF and HA‐CDK2 were transiently co‐transfected into HEK293T cells. Cells were cultured in media containing vehicle or 100 ng/ml CXCL12 (#460‐SD‐050, R&D Systems, Inc., MN, USA) for 24 h. Cells were lysed in a RIPA buffer containing a protease inhibitor cocktail (cOmplete™; #11697498001, Roche, IN, USA). Cell extracts were incubated with anti‐HA at 4°C overnight. Antibody‐bound proteins were precipitated with Dynabeads™ Protein G (10003D, Invitrogen, OR, USA). Samples were separated by SDS‐PAGE and transferred to PVDF membranes. The membrane was blocked for 1 h at room temperature and incubated with primary antibodies 4°C overnight. Membranes were washed three times with TBST and incubated with HRP‐conjugated secondary antibodies for 2 h. Blots were washed three times with TBST and developed with the ECL system according to the manufacturer's protocols. Primary antibodies are listed in Table S1.

### 2.8 Testis tissue culture

Testes from PN 1D WT and *Mast4* KO male mice were decapsulated and gently fragmented into several pieces 1–3 mm in diameter. Explants of testes were cultured as modified from the methods described by Sato et al.^29^ Briefly, the tissues were placed in a Trowell‐type organ culture and incubated with RPMI medium 1640 (#11875‐093, Life Technologies, USA) supplemented with 10% knockout serum replacement (#10828‐010, Life Technologies, USA), 100 units/ml of penicillin, and 100 μg/ml of streptomycin at 37°C in 5% CO_2_. The medium was added to bFGF (#100‐18B; PeproTech, Inc., USA) and CXCL12 (460‐SD‐010; R&D Systems, Inc., USA) (100 ng/ml final) or vehicle. The culture medium was replaced every 2 days. Explants of testes were collected 1 week later and fixed in 4% paraformaldehyde or harvested using TRIzol® reagent.

### 2.9 Statistical analysis

The graphic results were expressed as the mean ± SD. A GraphPad Prism 7 (GraphPad Software, San Diego, CA, USA) was used to analyse the data. Comparison of two groups was performed using an unpaired two‐tailed *t*‐test. Comparison of multiple groups was performed by one‐way ANOVA followed by Tukey's multiple comparisons test. The *p* value < 0.05 was considered significant.

## 3 RESULTS

### 3.1 Spermatogenic dysregulation and decreased PLZF expression in *Mast4* KO testes

**FIGURE 1:**
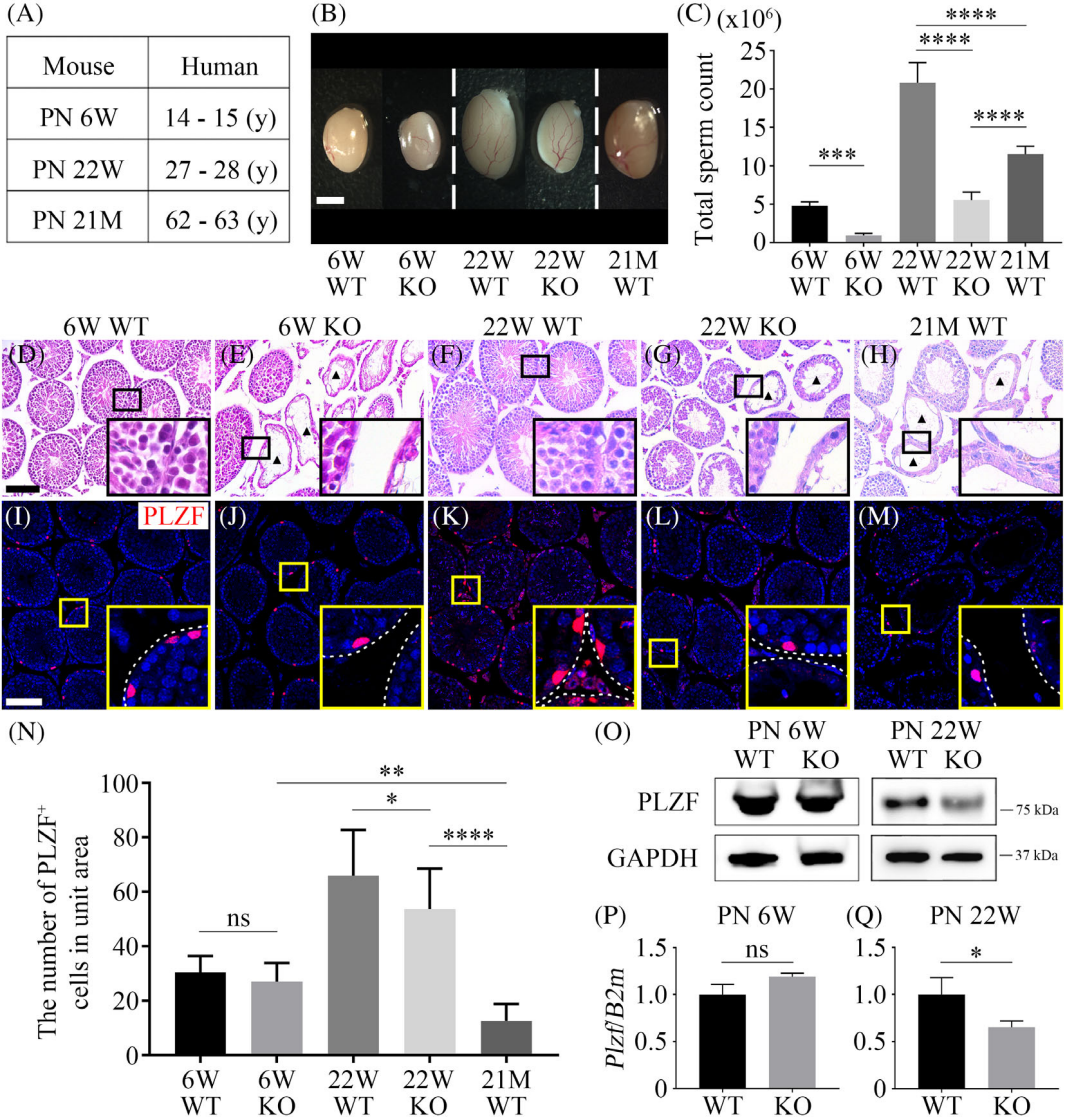
The dysfunction of spermatogenesis and decreased PLZF expression in *Mast4* KO testes. (A) Life history stages in C57BL/6 mice in comparison to those in human. (B) *Mast4* KO testes reduced in size compared to WT testes in both stages. The testes of PN 21M WT mice are larger than other *Mast4* KO testes. (C) The sperm count in *Mast4* KO testes decreased by a quarter compared to that in the WT. In PN 21M WT testes, sperm count is more than double of that in the KO testes. (D–H) HE staining of testes in WT and *Mast4* KO mice. WT testes from (D) PN 6W and (F) PN 22W mice have well‐organized seminiferous tubules. All types of germ cells are observed in the seminiferous tubules. KO testes from (E) PN 6W and (G) PN 22W mice have SCO tubules. Germ cells are depleted and only Sertoli cells are observed (arrowheads). (H) PN 21M mice testes have SCO tubules similar to those in *Mast4* KO testes (arrowheads). (I–M) Immunohistochemistry of PLZF in WT and *Mast4* KO testes. PLZF is localized in spermatogonial stem cells in the outermost layer of the seminiferous tubules. Compared to (I) PN 6W and (K) PN 22W WT testes, PLZF expression in (J) PN 6W and (L) PN 22W KO testes is decreased, respectively. (M) In PN 21M WT testes, PLZF expression is significantly decreased. (N) Quantification of PLZF expression in WT and *Mast4* KO testes (*n* = 15, five images per group of three mice). (O) Western blot of PLZF in WT and *Mast4* KO testes. (P, Q) RT‐qPCR of *Plzf* in WT and *Mast4* KO testes. (N–Q) Expression of PLZF is significantly decreased in PN 22W KO mice but not in PN 6W KO mice. Scale bars; B, 2 mm, D–M, 100 μm, *p < 0.05, **p < 0.01, ***p < 0.001, ****p < 0.0001, ns; no significance

The maturation rate of mice is 150 times faster during the first month of life and 45 times faster over the next 5 months than that of humans, during which mice pass through their mature adult stage.^30^ Previous studies have reported spermatogenic differences between wild‐type (WT) and *Mast4* KO mice in PN 6W,^26^ which correlates with the puberty stage of a 14–15‐year‐old human. To investigate the effect of *Mast4* deletion in mature adult stages, differences between PN 22W and postnatal month 21 (PN 21M) were analysed, which correlate with a 27–28‐year‐old and a 62–63‐year‐old human, respectively (Figure 1A). First, the size reduction was observed in the testes of PN 6W and PN 22W KO mice compared to those of corresponding WT mice. In addition, the testes of PN 21M WT mice were similar in size to those of PN 22W WT mice and were significantly larger than those of PN 6W testes (Figure 1B). Furthermore, sperm count decreased in KO testes of PN 6W and PN 22W mice. PN 21M mice also had a higher sperm count than those of PN 6W and PN 22W KO mice (Figure 1C). The weight, height and width of all testes were also compared; in PN 6W mice, all these characteristics were significantly lower in KO testes than in WT testes (Figure S2A–C). However, these changes were not significant in PN 22W testes (Figure S2D–F). In comparison with the WT, only the weight of PN 6W testes showed a significant difference (Figure S2G), although the height and width did not differ among stages (Figure S2H–I). Next, the testes were histologically examined by haematoxylin and eosin (H&E) staining and immunohistochemistry. In PN 6W mice, WT testes had well‐organized and compact seminiferous tubules (Figure 1D), while KO testes had germ cell‐depleted or Sertoli cell‐only (SCO) tubules (Figure 1E arrowheads). PN 22W WT testes also had seminiferous tubules in which all germs were observed and connected to each other (Figure 1F). In PN 22W KO testes, SCO tubules were observed and germ cell‐depleted tubules increased compared to those in PN 6W KO testes (Figure 1G arrowheads). PN 21M WT testes showed a phenotype similar to that of KO testes, with wide interstitial spaces and SCO tubules (Figure 1H arrowheads). In addition, PLZF expression was examined to determine alterations in SSCs in the seminiferous tubules of WT and KO testes. In PN 6W WT testes, PLZF was sparsely localized in the outermost layer of the seminiferous tubules (Figure 1I), whereas the expression of PLZF decreased in KO testes (Figure 1J). In PN 22W testes, PLZF expression increased overall compared to that in PN 6W testes, and KO testes showed decreased expression of PLZF compared to that in WT testes (Figure 1K,L). Interestingly, in PN 21M WT testes, PLZF expression was lower than that in other KO testes (Figure 1M). PN 6W mice showed no significant differences between WT and KO testes (Figure 1N). However, PLZF expression in PN 22W KO testes was significantly lower than in PN 22W WT testes. Furthermore, PN 21M WT testes had the lowest PLZF levels among all the experimental groups, showing significant decreases. In addition, western blot and RT‐qPCR analyses of PN 6W and PN 22W testes indicated that PLZF expression significantly decreased in PN 22W KO testes (Figure 1O–Q). Thus, *Mast4* deletion led to the depletion of germ cells and SCO tubules in the testes, similarly to aged (PN 21M) mice, and significantly decreased PLZF expression in the adult stage. It is inferred that spermatogenesis, especially SSC self‐renewal, is dysregulated in *Mast4* KO testes.

### 3.2 Cell cycle alteration of SSCs in *Mast4* KO testes

**FIGURE 2:**
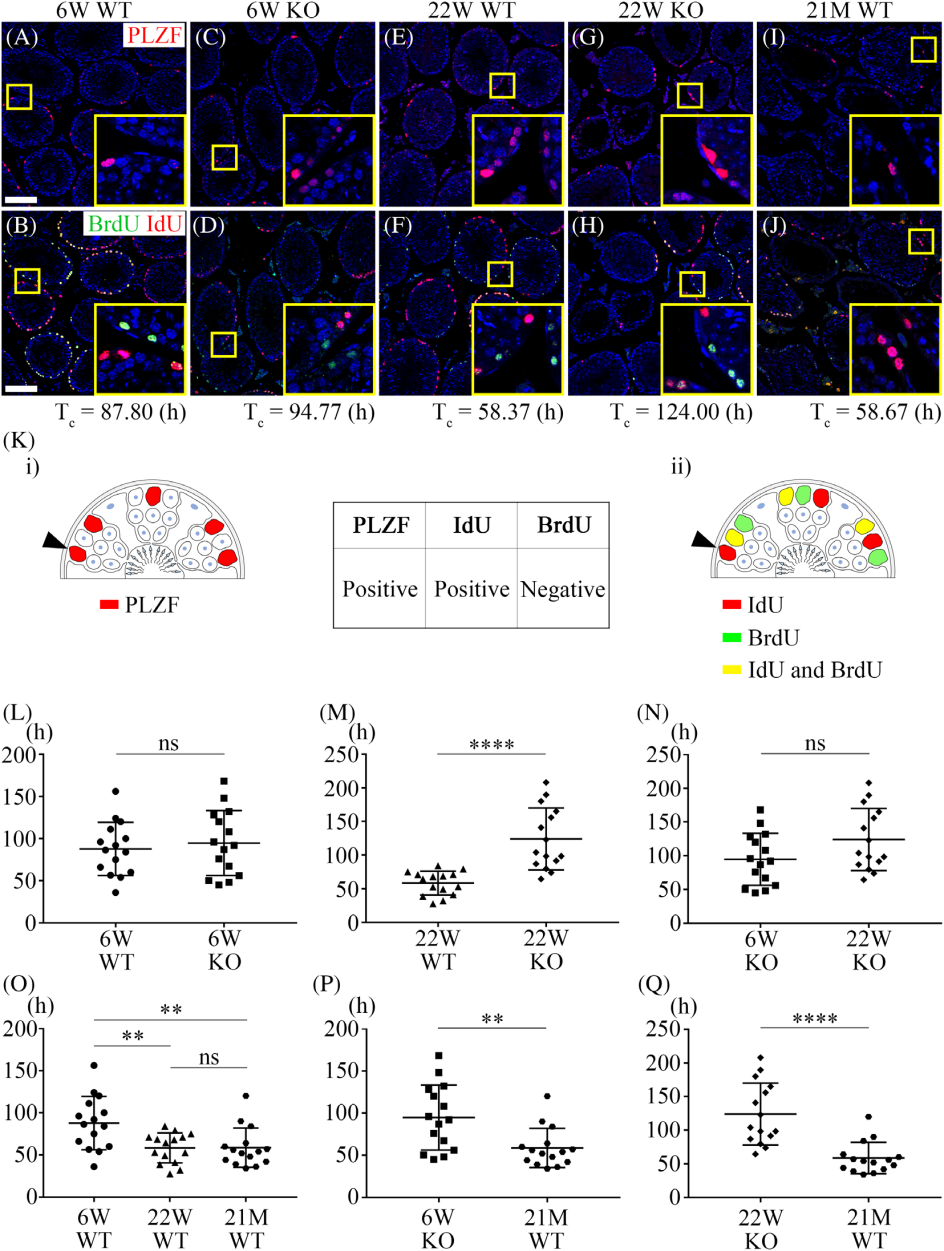
Analyses of *T*_*c*_ in SSCs between WT and *Mast4* KO testes. (A–J) Immunohistochemistry of IdU/BrdU and PLZF for cell cycle calculation in SSCs in WT and *Mast4* KO testes. *T*_*c*_ was calculated from the co‐localization of IdU‐only labelled and PLZF‐labelled cells by serial sectioning. (K) Schematic diagram of cell cycle calculation in IdU/BrdU staining with PLZF‐expressing SSCs. For immunohistochemistry of PLZF and IdU/BrdU by serial sectioning after IdU/BrdU injection, (K‐i) PLZF‐positive cells were counted per unit area (P_cells_, arrowhead). (K‐ii) In adjacent serial sectioning, IdU‐only labelled cells were counted under co‐localization with PLZF (L_cells_, arrowhead). *T*_*c*_ was calculated using the formula in Figure S1 for cell cycle. (L–Q) Statistical analysis *T*_*c*_ at each stage (*n* = 15). (L) *T*_*c*_ in PN 6W KO SSCs is similar to that in WT SSCs. (M) *T*_*c*_ dramatically increases in PN 22W KO SSCs compared to that in WT SSCs. (N) *T*_*c*_ differences are not shown between PN 6W and PN 22W KO SSCs. (O) Among WT groups, *T*_*c*_ in PN 6W SSCs is longer than that in other SSCs. PN 22W and PN 21M SSCs have similar *T*_*c*_ values. (P, Q) *T*_*c*_ in PN 21M WT SSCs is shorter than that in (P) PN 6W and (Q) PN 22W KO SSCs. Scale bars; 100 μm, **p < 0.01, ****p < 0.0001, ns; no significance

Since the self‐renewal of SSCs was not properly regulated in *Mast4* KO testes, their cell cycle was examined to understand the cellular mechanisms involved. Cell cycle time (*T*_*c*_) was calculated using IdU/BrdU injection at 12 h intervals in WT and *Mast4* KO testes. To label the SSCs, PLZF was also examined, and the co‐localization of IdU‐only‐labelled cells and PLZF‐labelled cells was checked in serial sections (Figure 2A–J). The average *T*_*c*_ values from the SSCs in each group were assumed to be 87.8 h (Figure 2A,B), 94.8 h (Figure 2C,D), 58.4 h (Figure 2E,F), 124 h (Figure 2G,H) and 58.7 h (Figure 2I,J). The whole raw *T*_*c*_ data are presented in Table S3. To calculate the cell cycle, PLZF‐positive cells were counted in one section slide, then IdU‐positive/BrdU‐negative cells were counted in adjacent serial section slide which co‐localized with PLZF (Figure 2K). IdU/BrdU injection and the formula for cell cycle calculation are described in detail in Figure S1. *T*_*c*_ was not significantly different between PN 6W WT and KO SSCs (Figure 2L); however, it significantly increased in PN 22W KO SSCs compared to that in the corresponding WT SSCs (Figure 2M). *T*_*c*_ of KO SSCs from both stages were not significantly different (Figure 2N). In PN 21M WT SSCs, the cell cycle was similar to that in PN 22W WT SSCs, although these SSCs had the lowest PLZF expression (Figure 2O). In addition, *T*_*c*_ in PN 21M WT SSCs was shorter than that in the KO SSCs (Figure 2P,Q). Taken together, *T*_*c*_ of SSCs increased not in pubertal stages but in adult stages of *Mast4* KO mice, indicating that cell cycle decreased in adult *Mast4* KO SSCs.

### 3.3 Effect of MAST4 on cell cycle in SSCs

**FIGURE 3:**
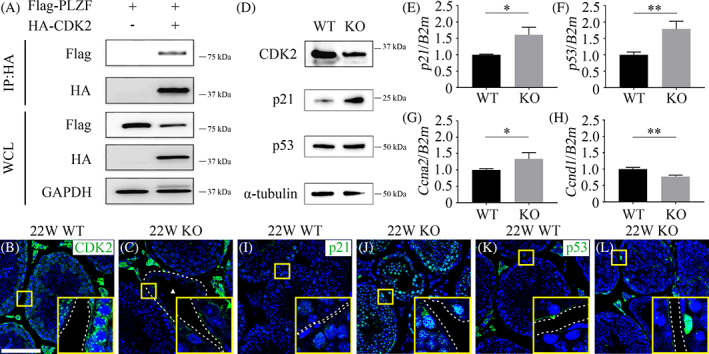
Effect of MAST4 on CDK2‐PLZF mechanisms. (A) Interaction between PLZF and CDK2 was examined. Flag‐PLZF and HA‐CDK2 were transiently co‐transfected into HEK293T cells, followed by HA pull‐down. Note the interaction between PLZF and CDK2. (B) In PN 22W WT testes, CDK2 is expressed in the cytoplasm of spermatogonia located in the outermost layer of seminiferous tubules. (C) In PN 22W *Mast4* KO testes, CDK2 expression significantly decreases compared to that in WT testes (arrowhead). (D) Western blot analysis of PN 22W WT and *Mast4* KO testes indicates that CDK2 expression decreases in PN 22W *Mast4* KO testes. The expression of p21 increases in PN 22W *Mast4* KO testes, while p53 levels does not significantly change. (E–H) RT‐qPCR analyses of PN 22W WT and *Mast4* KO testes indicate that the expression of (E) *p21*, (F) *p53* and (G) *Ccna2* significantly increases in PN 22W *Mast4* KO testes. (H) *Ccnd1* expression decreases in PN 22W *Mast4* KO testes. (I) In PN 22W WT testes, p21 is rarely expressed in seminiferous tubules. (J) In PN 22W *Mast4* KO testes, p21 expression significantly increases compared to that in WT testes. (K) In PN 22W WT testes, p53 is rarely expressed in seminiferous tubules. (L) In PN 22W *Mast4* KO testes, p53 expression slightly increases compared to that in WT testes. Scale bars; 100 μm, *p < 0.05, **p < 0.01

To determine how MAST4 regulates cell cycle in SSCs, the molecular mechanism of this protein was investigated. CDK2 is a key regulator of G1–S transition in stem cells by phosphorylating PLZF.^23^ An immunoprecipitation assay with Flag‐PLZF and HA‐CDK2 co‐transfected into HEK293T cells confirmed the interaction between PLZF and CDK2 (Figure 3A). In addition, the expression of CDK2 and PLZF target genes was examined in PN 22W WT and *Mast4* KO testes to determine whether *Mast4* deletion affects CDK2‐PLZF mechanisms in SSCs. While CDK2 was observed in the outermost layer of the seminiferous tubules in WT testes, depletion of *Mast4* reduced CDK2 expression (Figure 3B,C arrowhead). Western blotting of PN 22W WT and KO testes also showed that the expression of CDK2 decreased in KO testes (Figure 3D). It has been reported that PLZF binds to the promoter region of *p21*, *p53* and *Ccna2*, and represses their expression.^23^, ^25^ In KO testes, the expression of p21 significantly increased, and p53 expression was similar to that in WT testes (Figure 3D). Furthermore, RT‐qPCR data indicated that the expression of *p21*, *p53* and *Ccna2* significantly increased in KO testes (Figure 3E–G). The expression of *Ccnd1*, which represents cells in the G1 phase and G1‐S transition, decreased in KO testes (Figure 3H). Immunohistochemical analysis of p21 and p53 showed similar results (Figure 3I–L). Taken together, CDK2 directly interacts with PLZF, and MAST4 regulates the expression of CDK2 and PLZF, and subsequently, the transcriptional level of PLZF target genes.

### 3.4 FGF2‐MAST4‐CXCL12 pathway straightens disturbed cell cycle of SSCs in *Mast4* KO testes

**FIGURE 4:**
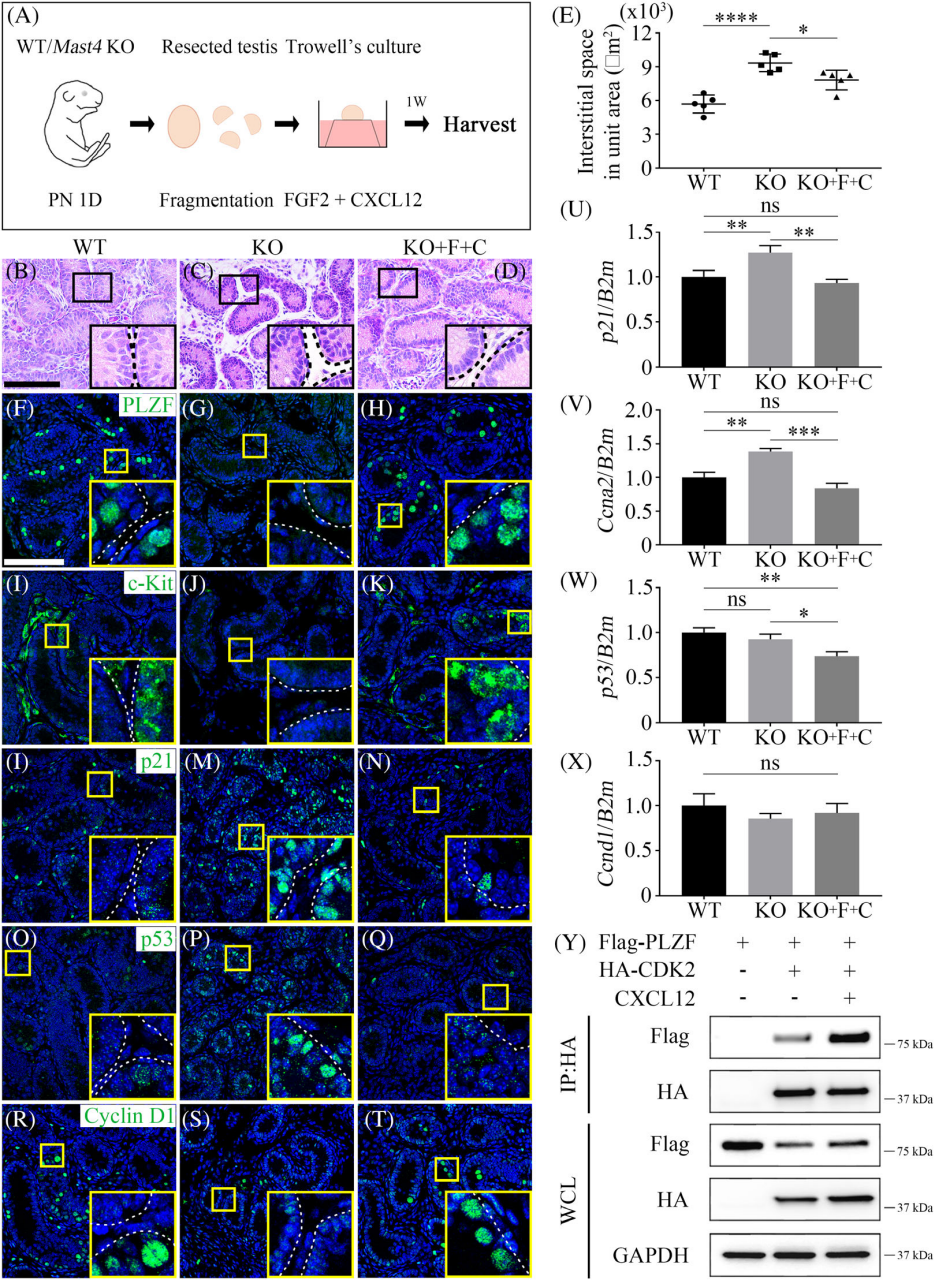
FGF2‐MAST4‐CXCL12 pathway modulates cell cycle in SSCs. (A) Schematic diagram for testes tissue culture procedure. (B–D) HE staining of PN 1D testes cultured for 1 week in vitro. (B) WT testes show well‐organized seminiferous tubules. (C) *Mast4* KO testes show irregular tubular structure and wider interstitial space compared to those in WT testes. (D) KO + F + C testes have similar seminiferous tubular structure compared to that in WT testes. (E) Quantitative analysis of interstitial space in testes tissue culture. KO testes show significantly widened area compared to that in WT testes. KO + F + C testes have decreased interstitial space. (F–T) Immunohistochemistry analyses of PN 1D testes cultured for 1 week. (F) In WT testes, PLZF is sparsely localized in the outermost layer of seminiferous tubules. (G) PLZF expression is not observed in KO testes. (H) In KO + F + C testes, PLZF expression is similar to that in WT testes. (I) In WT testes, c‐Kit localizes partially in the outermost layer of seminiferous tubules. (J) KO testes do not express c‐Kit. (K) Expression of c‐Kit is observed in KO + F + C testes. (L) p21 is expressed sparsely in the spermatogonia of seminiferous tubules in WT testes. (M) p21 expression dramatically increases in KO testes. (N) The expression of p21 in KO + F + C testes is similar to that in WT testes. (O) p53 is rarely expressed in seminiferous tubules in WT testes. (P) p53 expression significantly increases in KO testes. (Q) In KO + F + C testes, p53 expression is similar to that in WT testes. (R) Cyclin D1 is partially expressed in spermatogonia of WT testes. (S) Although the expression of cyclin D1 decreases in KO testes, (T) its expression in KO + F + C testes is similar to that in WT testes. (U) *p21* expression increases in KO testes and decreases in KO + F + C testes, similar to that in WT testes (*n* = 3). (V) *Ccna2* expression increases in KO testes and decreases in KO + F + C testes, similar to that in WT testes (*n* = 3). (W) While there is no significant difference in *p53* expression between WT and KO testes, *p53* expression significantly decreases in KO + F + C testes compared to that in the other groups (*n* = 3). (X) *Ccnd1* expression is not significant among all groups (*n* = 3). (Y) The effect of CXCL12 on PLZF and CDK2 interaction was examined. Flag‐PLZF and HA‐CDK2 were transiently co‐transfected into HEK293T cells, followed by CXCL12 treatment (100 ng/ml) for 24 h. CXCL12 increases the interaction between PLZF and CDK2. F; FGF2, C; CXCL12, WCL; Whole cell lysates. Scale bars; 100 μm. *p < 0.05, **p < 0.01, ***p < 0.001, ****p < 0.0001, ns; no significance

To determine whether the reduced cell cycle progression in SSCs could be rescued by the FGF2‐MAST4‐CXCL12 pathway,^26^ an in vitro tissue culture system was used. Postnatal day 1 (PN 1D) testes of WT and *Mast4* KO mice were fragmented and cultured. To induce the MAST4 pathway, exogenous FGF2 and CXCL12, which are upstream and downstream of MAST4, respectively, were added to the culture medium (Figure 4A). One week after culture, WT testes had well‐organized seminiferous tubular structures, as observed by H&E staining (Figure 4B). However, KO testes had an irregular spermatogonia structure and a wider interstitial space than WT testes did (Figure 4C). Exogenous FGF2 and CXCL12 rescued the enlarged interstitial space in KO testes, similar to that in WT testes (Figure 4D). Quantification of the interstitial space area indicated that the interstitial space of KO testes significantly increased compared to that of WT testes, and KO testes cultured with FGF2 and CXCL12 (KO + F + C) showed a decreased interstitial space area compared to that of KO testes (Figure 4E). The expression of SSC‐ and cell cycle‐related proteins was examined using immunohistochemistry. The expression of PLZF and c‐Kit, which is a marker of differentiating spermatogonia, decreased to the extent that it was invisible in KO testes and recovered in KO + F + C testes, similar to that in WT testes (Figure 4F–K). Subsequently, the expression of PLZF target genes, p21 and p53, was examined. Their expression is rarely observed in spermatogonia (Figure 4L,O); however, the expression of p21 and p53 significantly increased in KO testes and recovered in KO + F + C testes (Figure 4M,N,P,Q). Cyclin D1 was expressed in differentiating spermatogonia of WT testes (Figure 4R), whereas its expression decreased in KO testes. KO + F + C testes expressed cyclin D1, similar to WT testes (Figure 4T). These results were also supported by the RT‐qPCR analyses. The expression of *p21* and *Ccna2* increased in KO mice and recovered in KO + F + C testes (Figure 4U,V). Although *p53* expression did not significantly change in KO testes, it decreased in KO + F + C testes compared to that in other groups (Figure 4W). However, the expression of *Ccnd1* was not significant (Figure 4X). The interaction between PLZF and CDK2 increased after CXCL12 treatment (Figure 4Y). Taken together, the FGF2‐MAST4‐CXCL12 pathway is crucial for maintaining cell cycle in SSCs by regulating PLZF and its target gene expression.

## 4 DISCUSSION

Mammalian spermatogenesis is a classic adult stem cell‐based process that is regulated by mitosis and meiosis, and is supported by the self‐renewal and differentiation of SSCs. Studying SSCs provides a model to better understand adult stem cell biology, and deciphering the mechanisms that control SSC functions may lead to the treatment of male infertility. SSC self‐renewal is a necessary process for transferring gametes to the next generation, and the cell cycle of SSCs must be well maintained during this process. The present study suggests that MAST4 is involved in cell cycle maintenance and self‐renewal regulation of SSCs.

MAST4 harmoniously exerted its functions by regulating various properties during spermatogenesis in a stage‐dependent manner. In the pubertal stage (PN 6W), testis size and sperm count of KO mice decreased compared to those of WT mice; however, PLZF level did not significantly reduce. Notably, PLZF expression significantly decreased in adult (PN 22W) KO testes compared to that in WT testes. Therefore, the failure of SSC self‐renewal due to *Mast4* deletion just began or did not proceed significantly in the pubertal stage.

PLZF regulates proliferation, apoptosis and cell cycle in various cell types.^31^, ^32^, ^33^ *Plzf* KO mice exhibited agametic seminiferous tubules similar to those in *Mast4* KO mice.^34^ As *Mast4* KO and *Plzf* KO testes shared the same morphology, mechanisms regulating SSC self‐renewal are expected to also have common features. In the present study, SSC cell cycle significantly decreased in adult *Mast4* KO testes, suggesting that MAST4 controls the cell cycle and self‐renewal of SSCs by regulating PLZF expression.

CDK2, which regulates cell cycle especially in the G1 phase, has been known to interact with PLZF and suppress the transcription of *Ccna2*.^23^, ^35^ Although PLZF has been extensively studied in various animal models as a marker of SSCs,^34^, ^36^, ^37^, ^38^ CDK2 has rarely been investigated in SSCs,^39^ and its role has been studied mostly in meiosis.^40^, ^41^, ^42^ It was difficult to accurately investigate the regulation of the interaction between PLZF and CDK2 because MAST4 is expressed and functions in Sertoli cells and only transmits a signal to SSCs. To overcome this problem, the expression of CDK2 and PLZF in *Mast4* KO testes and the effect of CXCL12 on the interaction between CDK2 and PLZF were examined. Because CXCL12 is activated and then transmits the paracrine signal from Sertoli cells to SSCs via the FGF2‐MAST4‐ERM pathway.^26^ CDK2 expression decreased in adult *Mast4* KO testes, whereas the interaction between CDK2 and PLZF increased following CXCL12 treatment.

A tissue culture system was introduced to regulate the mechanisms of various cell populations in the testis that cannot be resolved in cell culture. Through in vitro tissue culture of the testes, irregular tubular structures and widened interstitial spaces were observed in *Mast4* KO mice, which were recovered by both FGF2 and CXCL12. In addition, the expression of SSC‐related proteins, such as PLZF and c‐Kit, was downregulated in *Mast4* KO testes. So, it was examined whether the reduced cell cycle progression can be restored through the MAST4 pathway. The function of PLZF in KO + F + C testes was similar to that in WT testes, as shown by immunohistochemistry and RT‐qPCR analysis of PLZF target genes. Therefore, it can be inferred that the SSC cell cycle is regulated within the signalling pathway in which MAST4 operates.

**FIGURE 5:**
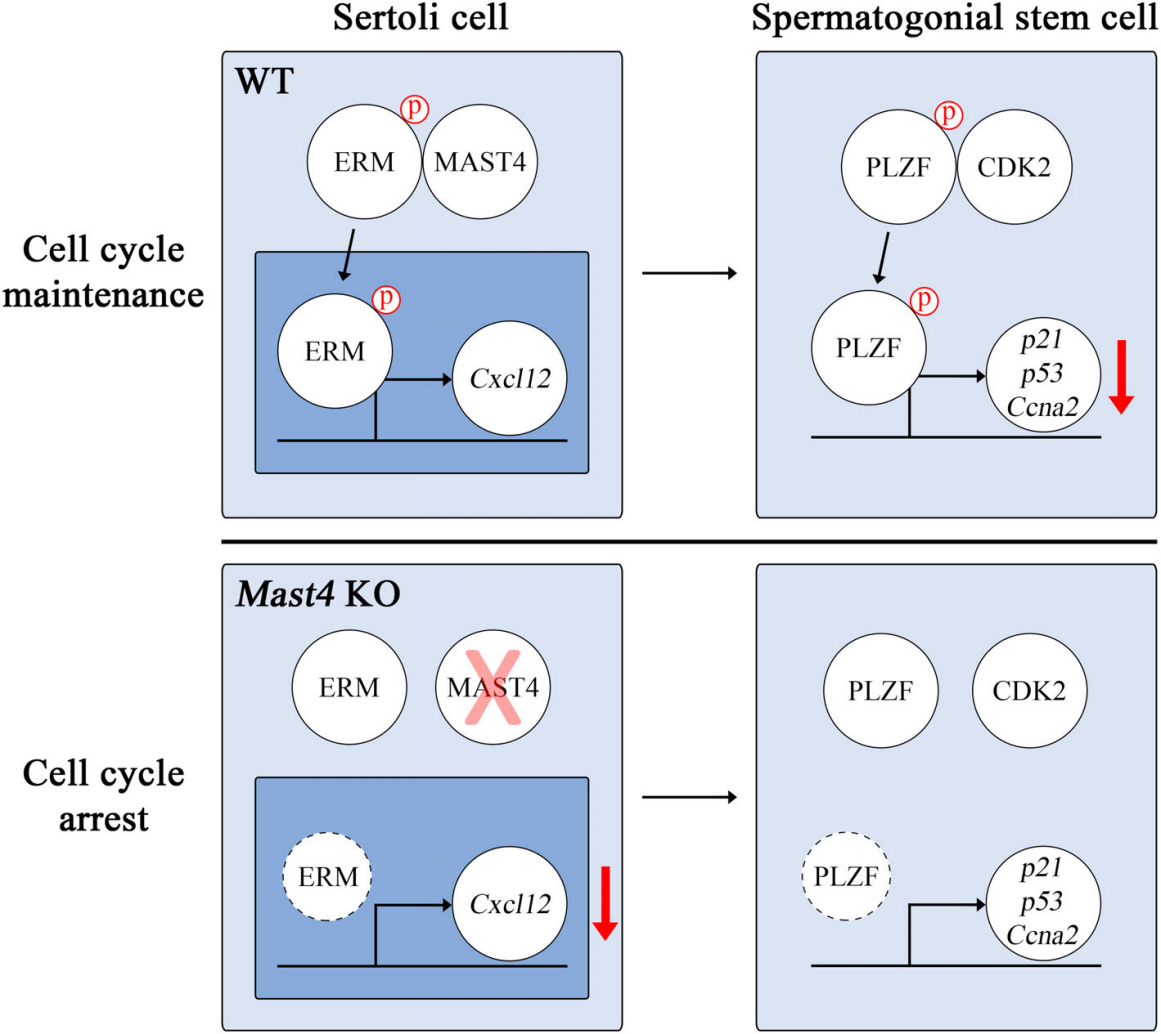
Maintenance of cell cycle in SSCs through MAST4‐PLZF mechanism. MAST4 phosphorylates ERM and subsequently regulates the transcription of *Cxcl12*, which is the target gene of ERM in Sertoli cells. CXCL12 migrates to SSCs and transmits signals involved in SSC self‐renewal. PLZF is phosphorylated by CDK2 and suppresses the transcription of *p21*, *p53* and *Ccna2*. Inhibition of *Ccna2* transcription enables SSCs to maintain their cell cycle. *Mast4* KO decreases the transcription of *Cxcl12* and interaction between PLZF and CDK2. Inhibition of PLZF could not suppress the transcription of its target genes, leading to cell cycle arrest in SSCs.

In conclusion, MAST4 is closely related to the maintenance of SSCs by regulating their cell cycle (Figure 5). In Sertoli cells, MAST4 phosphorylates ERM and subsequently regulates the transcription of *Cxcl12*, which is the target gene of ERM. CXCL12 migrates to SSCs and transmits signals involved in SSC self‐renewal. PLZF is phosphorylated by CDK2 and suppresses the transcription of *p21*, *p53* and *Ccna2*. Inhibition of the transcription of PLZF target genes enables SSCs to maintain their cell cycle. Taken together, this study provides new insights into a novel mechanism by which MAST4 regulates CDK2 and PLZF involved in SSC self‐renewal. Moreover, our findings not only suggest the significance of apprehending spermatogenesis but also reflect its potential therapeutic usage in spermatogenic dysregulation.

## AUTHOR CONTRIBUTIONS

Seung‐Jun Lee contributed to design, data acquisition, analysis, interpretation and drafted; Ka‐Hwa Kim and Dong‐Joon Lee contributed to analysis, interpretation, critically revised the manuscript; Pyunggang Kim, Jinah Park and Seong‐Jin Kim contributed to interpretation and critically revised the manuscript; Han‐Sung Jung contributed to conception, and interpretation, critically revised the manuscript. All authors gave final approval and agreed to be accountable for all aspects of the work.

## FUNDING INFORMATION

This study was supported by the National Research Foundation of Korea (NRF) Grant funded by the Korea Government (MSIP) (NRF‐2022R1A2B5B03001627 and NRF‐2016R1A5A2008630).

## CONFLICT OF INTEREST

The authors declare no competing interest.

## Supporting information


**Data S1.** Supporting Information

## Data Availability

Data sharing is not applicable to this article as no new data were created or analyzed in this study.
