# Supplementary material for: MAST4 controls cell cycle in spermatogonial stem cells
Source: Cell Prolif. 2023 Jan 2;56(4):e13390. doi: 10.1111/cpr.13390 (PMC10068930; doi:10.1111/cpr.13390)
Supplement: Supplementary file 1 — Data S1. Supporting Information [file CPR-56-e13390-s001.docx]

**MAST4 controls cell cycle in spermatogonial stem cells**

**Seung-Jun Lee | Ka-Hwa Kim | Dong-Joon Lee | Pyunggang Kim | Jinah Park | Seong-Jin Kim | Han-Sung Jung**

**SUPPLEMENTARY TABLES AND FIGURES**

**Table S1. Antibodies for Immunohistochemistry (IHC), Western blot (WB) and Immunoprecipitation (IP)**

 **Table S2. Primers for RT-qPCR**

**
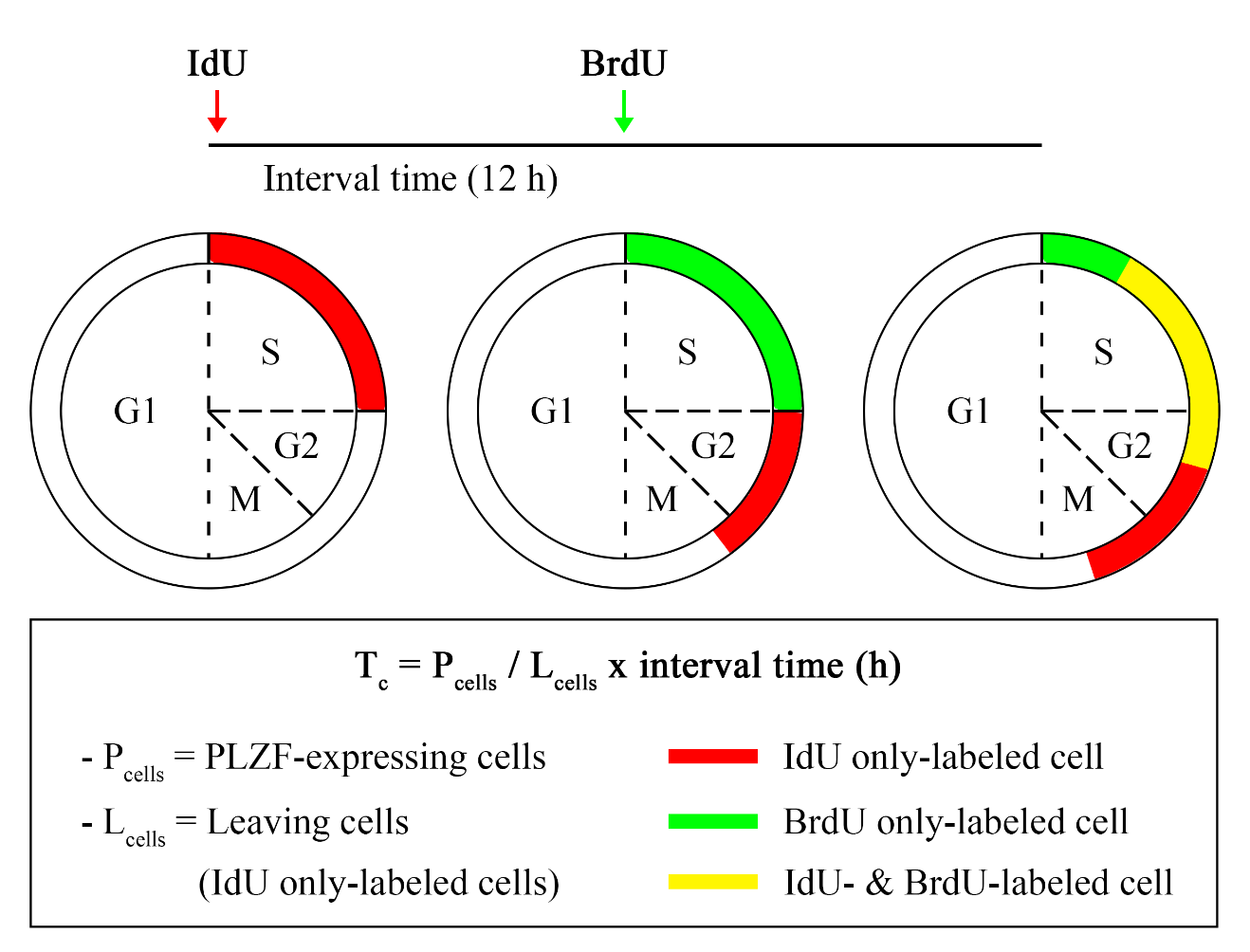
**

**Figure S1. IdU/BrdU injection for cell cycle calculation**

To estimate cell cycle time, the mice are injected with IdU at T = 0 h to label all cells in S phase at the beginning of the experiment. At T = 12 h, an injection of BrdU is given and the testes are fixed after 0.5 h, sufficient to label the S fraction at the end of the labeling period. During the 12 h interval when cells are exposed to IdU but not BrdU, some cells of the initial S phase will leave S-phase and consequently will be labeled just with IdU; this is the leaving fraction (L_cells_). By staining tissue sections with antibodies that allow to distinguish cells labeled with just IdU from those which incorporated BrdU and IdU, we can count the L_cells_ fractions.

**
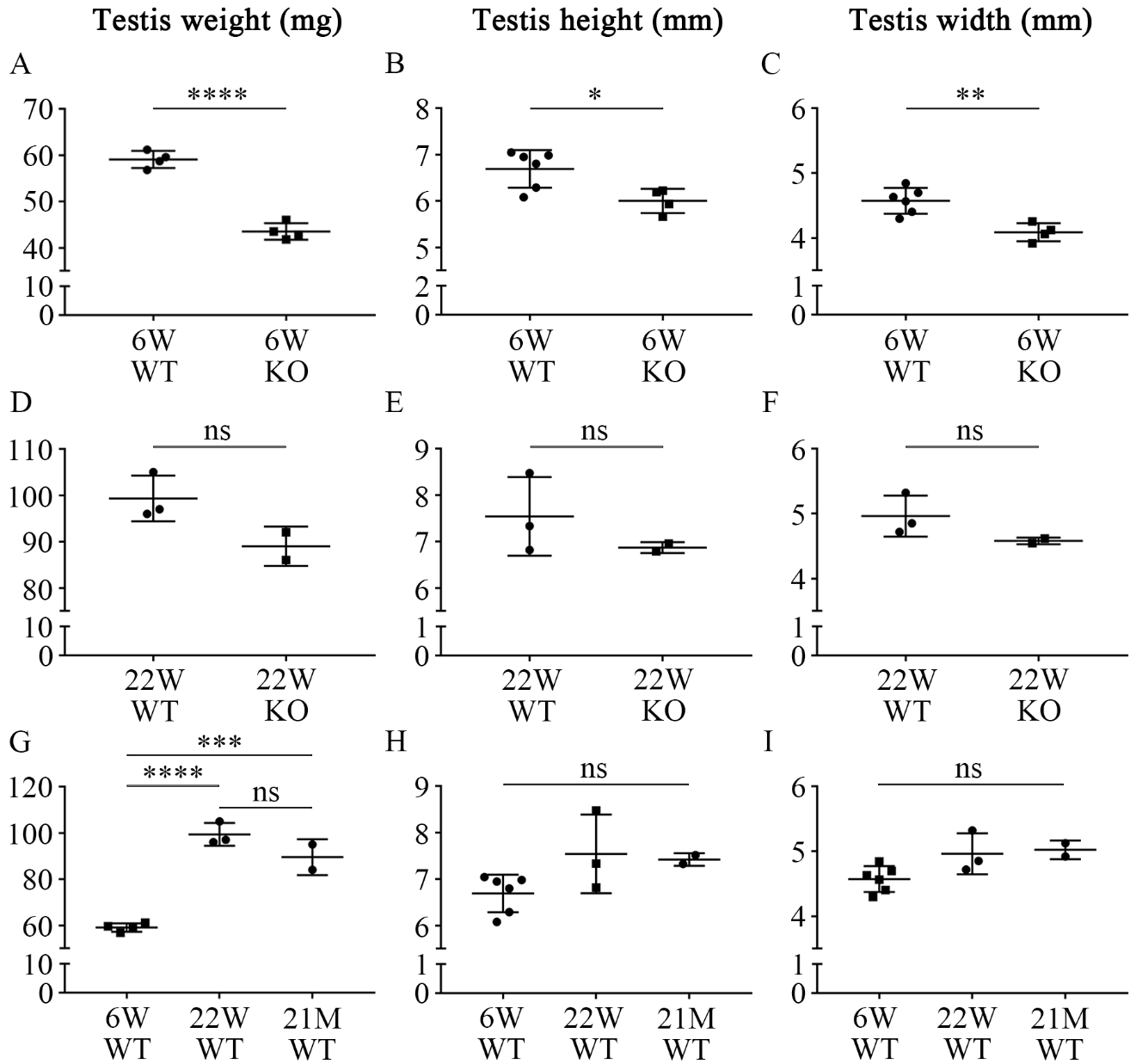
**

**Figure S2. Analyses of characteristics between WT and *Mast4* KO testes**

The characteristics of testes were analyzed and compared between WT and *Mast4* KO testes in (A–C) PN 6W and (D–F) PN 22W, as well as among WT testes in (G-I) PN 6W, 22W and 21M. In PN 6W, (A) weight, (B) height, and (C) width in *Mast4* KO testes are smaller than those in WT. The characteristics of PN 22W testes are not significantly different in (D) weight, (E) height and (F) width. (G) Weight of PN 6W testes is lower than that of other testes. (H) Height and (I) width of all WT testes are not significantly different. *p < 0.05, **p < 0.01, ***p < 0.001, ****p < 0.0001, ns; no significance.

**
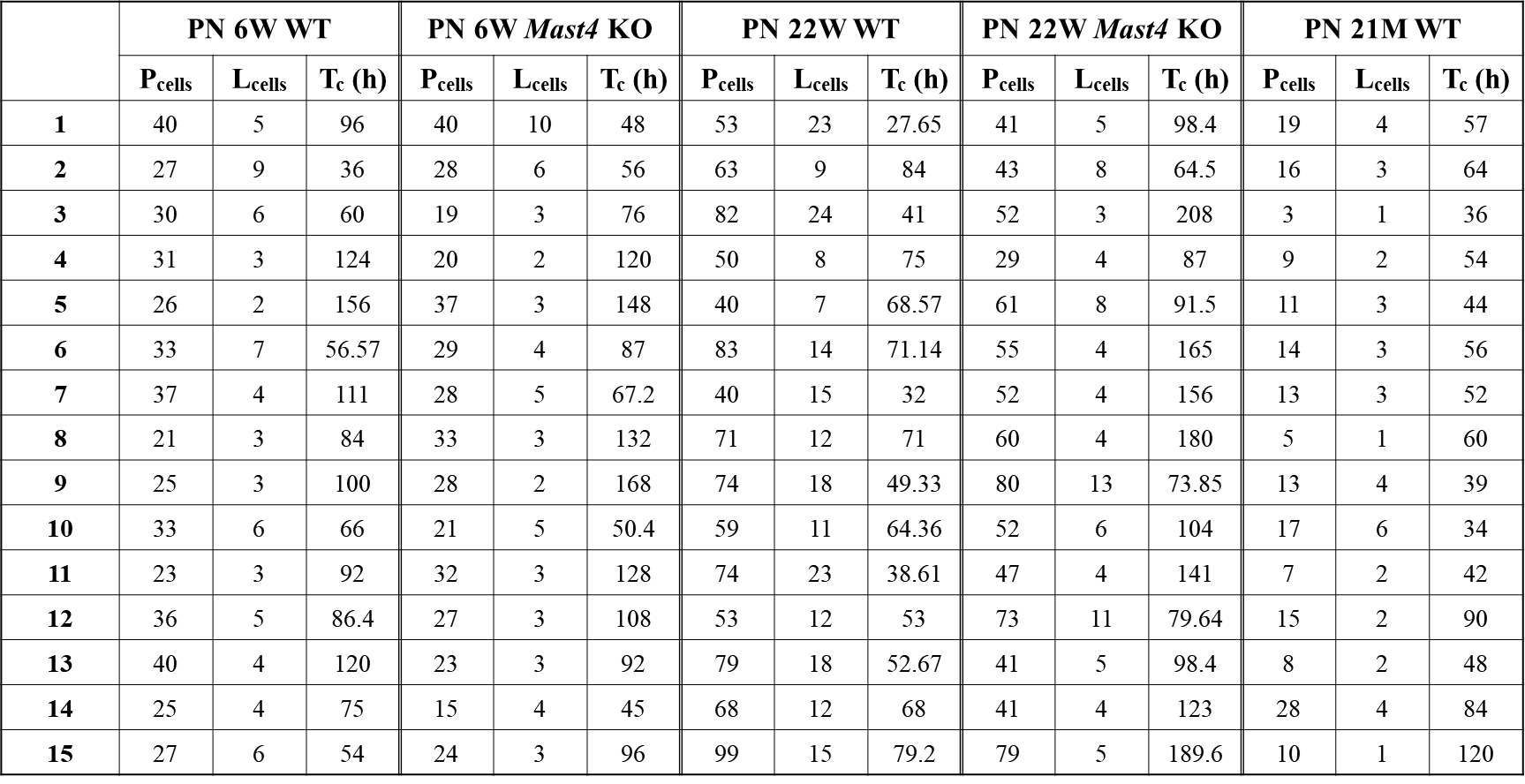
**

**Table S3. Quantitative data for cell cycle calculation of SSCs after IdU/BrdU injection to WT and *Mast4* KO mice.**

Immunohistochemical analyses of IdU/BrdU and PLZF in IdU/BrdU-injected WT and Mast4 KO testes (n = 15). T_c_ in PN 6W KO SSCs is similar to that in WT SSCs. T_c_ significantly increases in PN 22W KO SSCs compared to that in WT SSCs. T_c_ differences are not shown between PN 6W and PN 22W KO SSCs. T_c_ in PN 21M WT SSCs is shorter than that in PN 6W and PN 22W KO SSCs.
